# Are There Spillover Effects from the GI Bill? The Mental Health of Wives of Korean War Veterans

**DOI:** 10.1371/journal.pone.0154203

**Published:** 2016-05-17

**Authors:** Anusha M. Vable, Ichiro Kawachi, David Canning, M. Maria Glymour, Marcia P. Jimenez, S. V. Subramanian

**Affiliations:** 1 Stanford Prevention Research Center, Stanford University, Stanford, CA, United States of America; 2 Department of Social and Behavioral Sciences, Harvard T. H. Chan School of Public Health, Boston, MA, United States of America; 3 Department of Global Health and Populations, Harvard T. H. Chan School of Public Health, Boston, MA, United States of America; 4 Department of Epidemiology & Biostatistics, University of California San Francisco, San Francisco, CA, United States of America; 5 Department of Epidemiology, Brown University School of Public Health, Providence, RI, United States of America; National Center of Neurology and Psychiatry, JAPAN

## Abstract

**Background:**

The Korean War GI Bill provided economic benefits for veterans, thereby potentially improving their health outcomes. However potential spillover effects on veteran wives have not been evaluated.

**Methods:**

Data from wives of veterans eligible for the Korean War GI Bill (N = 128) and wives of non-veterans (N = 224) from the Health and Retirement Study were matched on race and coarsened birth year and childhood health using coarsened exact matching. Number of depressive symptoms in 2010 (average age = 78) were assessed using a modified, validated Center for Epidemiologic Studies-Depression Scale. Regression analyses were stratified into low (mother < 8 years schooling / missing data, N = 95) or high (mother ≥ 8 years schooling, N = 257) childhood socio-economic status (cSES) groups, and were adjusted for birth year and childhood health, as well as respondent’s educational attainment in a subset of analyses.

**Results:**

Husband’s Korean War GI Bill eligibility did not predict depressive symptoms among veteran wives in pooled analysis or cSES stratified analyses; analyses in the low cSES subgroup were underpowered (N = 95, β = -0.50, 95% Confidence Interval: (-1.35, 0.35), p = 0.248, power = 0.28).

**Conclusions:**

We found no evidence of a relationship between husband’s Korean War GI Bill eligibility and wives’ mental health in these data, however there may be a true effect that our analysis was underpowered to detect.

## Introduction

There is a robust relationship between low socio-economic status and psychological distress in individual studies [1–7] and in meta analyses [8]. There is further evidence that this relationship starts early in the lifecourse [1], and that upward social mobility is associated with better mental health [3], while downward social mobility is associated with worse mental health [9,10]. One hypothesized mechanism from SES to depression is through socially patterned stressors, whereby disadvantaged individuals are more likely to encounter stressors, and have fewer economic and social coping resources to deal with these stressors [11]. Inability to cope with stress may directly lead to feelings of hopelessness and depression [12], or may indirectly cause higher levels of depression through worse physical health [13].

This study examines if eligibility for a policy that facilitated upward social mobility had spillover effects to the wives of the intended recipients. We focus on husband’s eligibility for the Korean War GI Bill, formally called the “Veterans Readjustment and Assistance Act of 1952”, detailed in Box 1. The Korean War GI Bill (which offered similar benefits as the World War II and Vietnam war GI Bills) was designed to help veterans re-enter civilian life; these policies also resulted in increased socio-economic status (SES) for veterans in adulthood [14–21]. Further, recent analyses found that eligibility for Korean War GI Bill benefits is associated with a reduction of socio-economic disparities in depression markers for veterans compared to non-veterans [22].

**Box 1. Korean War GI Bill Benefits and Usage.**

The Korean War was a conflict between the Democratic People’s Republic of Korea (North Korea) and the Republic of Korea (South Korea), that occurred between 1950–1953 [27]. The “Veterans’ Readjustment and Assistance Act of 1952” aided American Korean War veterans with readjustment to civilian life. The bill provided educational subsidies, a government-backed low-interest loan benefit, unemployment benefits ($26 per week for 26 weeks), and mustering out pay ($100–$300) [28]. The educational subsidy applied to all veterans who served 90(+) days and were discharged other than dishonorably. Veterans were allowed money for education and training for 1.5 times the duration of service for up to 36 months of schooling. The amount of benefit varied by enrollment status and number of dependents, with a maximum of $160 per month for full-time students with more than one dependent [28]. Depending on the university and number of dependents, this subsidy cut college costs by 39–71% [16]. Of the total Korean War era veteran population of 5.5 million, the US government spent $4.5 billion ($30.8 billion in 2008 dollars) to educate 2.4 million veterans [29].

The GI Bill was a monumental social policy that democratized education for racial and religious minorities, however, very little is known about the spillover effects of the GI Bill beyond eligible veterans. We found one paper that examined children of likely World War II GI Bill recipients, and found veterans’ children were less likely to repeat a year of schooling [23]. We found no papers examining the effect of the GI Bill on wives of veterans. However, there are strong theoretical reasons to believe wives of Korean War veterans may benefit from their husband’s GI Bill eligibility.

The GI Bills have been previously been linked to increases in education [14–16], income [17,18,20,21], occupational status [18–21], and wealth [24]. While increases in education and occupational status may not directly spillover to wives of veterans, increases in income and wealth can directly benefit the whole household. We hypothesized that these increases in household SES are likely to decrease contact with stressors, and increase resources for dealing with stressors encountered, resulting in fewer depressive symptoms for veteran wives compared to non-veteran wives.

Further, we hypothesized differential effects of eligibility for the GI Bill in different socio-economic subpopulations. Among veterans, racially and socio-economically marginalized groups disproportionately benefited from military service [17–20]; similarly, we hypothesized that economic benefits from GI Bill eligibility would disproportionately benefit wives from socio-economically disadvantaged backgrounds, resulting in fewer depressive symptoms. Among women from high SES backgrounds, however, we hypothesize that GI Bill eligibility will have little or no impact.

We use three methodologic techniques to examine the relationship between husband’s Korean War GI Bill eligibility and wives’ mental health: a) covariate adjustment (CA), an approach frequently used in public health research, b) propensity score matching (PSM), a technique often used by epidemiologists, and c) coarsened exact matching (CEM), our main analytic approach, which is gaining traction in other social science disciplines, and posits better covariate balance between the treatment and control groups than PSM, and therefore a better approximation of a randomized controlled trial [25,26]. We compare the point estimates from all three methodologic approaches.

This paper advances the literature in three distinct ways. First, we investigate the spillover effects of eligibility for the Korean War GI Bill on the mental health of veterans’ wives, a population that has not been analyzed previously. Second, we examine these associations in socio-economic sub-populations to determine if particular groups were able to leverage these benefits more effectively than others. Third, we control rigorously for confounding in our main analytical approach, coarsened exact matching (CEM), detailed below, and compare results from CEM with results from propensity score matching (PSM), and covariate adjustment (CA) analytic techniques.

## Methods

### Sample

Data come from the Health and Retirement Study (HRS), a longitudinal, biennial sample of community-dwelling individuals 50 years of age and older and their spouses. Analyses were restricted to US-born women who reported marriage to a Korean War GI Bill eligible veteran or non-veteran in 2010; only wives of Korean War era veterans and wives of non-veterans were included in this analysis (i.e. wives of veterans from other time periods were excluded). We included women who were married to the same spouse since 1955, the year eligibility for the Korean War GI Bill ended, in an effort to minimize assortative mating by SES following receipt of GI Bill benefits. Of a total eligible sample of 863 women, 411 were excluded due to missing outcome data, leaving 452 (52.4%) women in the potential analytic sample. Additional individuals were pruned while implementing the coarsened exact matching procedure (CEM), detailed below, resulting in a final analytic sample of 352 women, 95 in the low childhood socio-economic status (cSES) group and 257 in the high cSES group.

### Exposure

Estimated eligibility for husband’s Korean War era GI Bill benefits was operationalized as any military service between 1950–1954; similar to other researchers investigating social policies [30,31], we are not able to separate eligible individuals from those who used the benefits; this approach will bias point estimates towards the null, analogous to an intent-to-treat analysis.

### Outcome

Depressive symptoms over the previous week were assessed with a modified 8-item Center for Epidemiologic Studies Depression (CESD) scale summing 6 “negative” items and two reverse-coded “positive” items (all coded yes / no); this scale is reliable among HRS participants (Cronbach’s alpha = 0.78) [32]. The negative items include feeling sad and depressed, everything is an effort, sleep is restless, feeling alone, and not able to get going, while the positive items asked if the respondent felt happy and enjoyed life; higher CESD scores indicate more depressive symptoms. The modified scale ranges from 0 to 8 and correlates with the original, 20-item scale [33]. Trained survey interviewers administered the questionnaire over the phone to a random half of the HRS sample, while the other half were administered at the respondent’s home. An indicator for elevated depressive symptoms was created by dichotomizing the 8-item measure at the HRS recommended cutoff of ≥ 4 symptoms [33].

We also conducted analyses on three markers of adult SES, educational attainment, household income per capita, and household wealth per capita. Educational attainment was reported as years of completed education (0–17+). Income and wealth in 2010 were adjusted for household size by dividing by the square root of number of household members.

### Effect Modification

Childhood socio-economic status (cSES) is evaluated as an effect modifier in this analysis. Based on prior work showing that mother’s education is a stronger predictor of adult outcomes than father’s education [34], we operationalized cSES as mother’s educational attainment, however in sensitivity analyses we also examined cSES based on father’s education to see if results are robust to different specifications. Parental education was recorded as < 8 years (low) or ≥ 8 years (high). Individuals with missing information on parents’ education were thought to have a distinct family structure (e.g. grew up in a single-parent household [35]), and were therefore included in the low cSES group, similar to other HRS researches examining cSES [3]. The same dichotomization criterion was used for father’s education.

### Matching Variables

Women were matched on birth year (3-year intervals for matching), cSES, race (Non-Hispanic White, Non-Hispanic Black, Hispanic), childhood self-rated health (dichotomized to excellent, very good vs. good, fair and poor for matching), history of depression during childhood; because birth year and childhood health were coarsened to facilitate matching, continuous birth year and indicator variables for childhood health (excellent health was the reference group) were added to the regression models described below. In supplemental analyses, we found veteran wives completed more education than non-veteran wives; therefore, educational attainment (recorded as 0–17 years) was included in a subset of the regression models as a linear spline with a knot at 12 years, a discontinuity at 16 years [36], and an indicator for those who completed a general education development (GED) test. The respondent’s education was included as a regression covariate rather than a matching variable because coarsened exact matching (CEM) specifies only pre-exposure covariates should be matched upon, and it is not clear if women completed their education before or after marriage.

The comparison analytic approaches, covariate adjustment (CA) and propensity score matching (PSM), included age in 2010 (linear, quadratic, and cubic terms), cSES, indicators for race (Non-Hispanic Whites were the reference group), indicators for childhood self-rated health (excellent health was the reference group), and an indicator for childhood depression. In CA models, all control variables were included in the model with the exposure simultaneously; in PSM models, these variables were used to predict the propensity of exposure.

### Analysis

The main analyses are performed using coarsened exact matching (CEM), a technique that matches treatment and control observations on pre-exposure covariates. Non-matches are pruned from the data set, and control units in the matched sample are weighted to create balance across stratum (i.e. treatment units are given a weight of one and control units are weighted to equal the number of treated units divided by the number of control units in the stratum, normalized to the total matched sample [25]). In the resultant analytical sample, the multivariate distribution of matching covariates is balanced between the treatment and control groups, mimicking a randomized control trial. CEM allows for matching on values as well as missing data [37]. Because both treatment and control units are pruned from the data set, the effect estimate is interpreted as the local sample average treatment effect among the treated. Quality of the analytic sample created by the CEM procedure is assessed with a multivariate imbalance measure, L_1_, which ranges from 0 (perfect overlap between treatment and control units on the multivariate distribution of matching covariates) to 1 (no overlap between treatment and control units) [37].

Two models are presented for each outcome after the matching algorithm was implemented: Model 1 uses the CEM weights, and represents the model used in the interaction analysis; Model 2 builds on Model 1 by adding a linear spline for the respondent’s educational attainment. An interaction analysis (wife of eligible veteran * cSES) was performed to determine if benefit eligibility had statistically different effects in low and high cSES groups. All models adjusted for birth year and self-reported childhood health as these variables were coarsened to facilitate matching. Linear regression models were used to calculate the average difference in depressive symptoms for wives of veterans and non-veterans and logistic regression was used to calculate the odds of elevated depressive symptoms. In addition to CEM, analyses were performed using a covariate adjustment approach (CA), and propensity score matching (PSM); a linear probability model is used to predict elevated depressive symptoms for all methodological techniques in the comparison analyses because this is the approach used by the teffects command in Stata 13 even when the outcome is dichotomous. PSM models used a logit model to predict the probability of exposure, and treated and control units were matched using one-to-one nearest neighbor matching with replacement. All data cleaning was performed in SAS, version 9.3, and analyses were performed in Stata, version 13.

### Ethics Statement

Health and Retirement Study data are collected by the University of Michigan, and the study was approved by the University of Michigan's Health Sciences Human Subjects Committee. These specific analyses were determined exempt by Harvard School of Public Health Office of Human Research Administration.

## Results

Wives of veterans and non-veterans included in this analysis were between 77 and 80 years old, majority white, the majority reported excellent or very good childhood health, and they reported very low levels of childhood depression (Table 1). Distributional differences in matching covariates between veteran and non-veteran wives were sharply reduced after implementing the CEM procedure; in the pooled population, the L_1_ decreased from 0.60 to 2.34*10^−16^ after the CEM algorithm was run, and similar reductions were observed in low and high cSES subgroups.

**Table 1:** Distribution of covariates in the CEM analytic sample.

		Low cSES (N = 95)				High cSES (N = 257)			
		Non-Veteran Spouses (N = 62)		Veteran Spouses (N = 33)		Non-Veteran Spouses (N = 162)		Veteran Spouses (N = 95)	
		N (mean)	% (sd)	N (mean)	% (sd)	N (mean)	% (sd)	N (mean)	% (sd)
Age in 2010		(80.0)	(3.9)	(76.7)	(3.4)	(79.1)	(3.1)	(76.8)	(1.8)
Non-Hispanic White		56	90.3	27	81.8	155	95.7	90	94.7
Non-Hispanic Black		4	6.5	4	12.1	6	3.7	4	4.2
Hispanic		2	3.2	2	6.1	1	0.6	1	1.1
Missing data on mother’s education		22	35.5	11	33.3	0	0	0	0
Missing data on father’s education		19	30.6	7	21.2	14	8.6	5	5.3
Childhood Health									
	Excellent	36	58.1	14	42.4	83	51.2	43	45.3
	Very good	17	27.4	13	39.4	58	35.8	32	33.7
	Good	6	9.7	4	12.1	15	9.3	10	10.5
	Fair	1	1.6	1	3.0	3	1.9	9	9.5
	Poor	2	3.2	1	3.0	3	1.9	1	1.1
	Depression	1	1.6	1	3.0	0	0	0	0

Spouses Korean War era veterans were exactly matched to spouses of non-veterans on all pre-exposure variables. The number of observations differs across variables because the coarsened exact matching (CEM) procedure allows for matching on missing data, however complete information is required for the exposure and the outcome variables; variables with missing data are indicated by an additional row. Although individuals are exactly matched, the distribution of variables may vary within cSES strata, however all covariates are equally distributed in the analysis sample after the CEM weights are applied.

There was no difference in number of depressive symptoms among wives of veterans and non-veterans in the pooled analysis or among the low or high cSES subgroups, however all point estimates were negative, suggesting veteran wives may have experienced fewer depressive symptoms than non-veteran wives; results were similar after additional adjustment for the respondent’s educational attainment (Table 2). Interaction analyses revealed the relationship between husband’s GI Bill eligibility and number of depressive symptoms did not vary by childhood SES (β = 0.52, 95%CI: (-0.38, 1.42), p = 0.259).

**Table 2:** Results for number of depressive symptoms among GI Bill eligible veteran wives compared to non-veteran wives.

	Model 1			Model 2		
	Beta	95% CI	p	Beta	95% CI	p
Whole Population	-0.23	(-0.62, 0.16)	0.253	-0.11	(-0.51, 0.29)	0.579
Low cSES	-0.50	(-1.35, 0.35)	0.248	-0.40	(-1.29, 0.50)	0.381
High cSES	-0.10	(-0.55, 0.35)	0.667	0.05	(-0.40, 0.51)	0.816

Model 1: model includes birth year (continuous) and self-rated childhood health (indicator variables for very good, good, fair poor) because these variables were coarsened to facilitate matching

Model 2: Model 1 with additional adjustment for respondent’s education (linear spline)

In the model for the whole population, CEM weights range from 0.07 to 5.01; in the low cSES population, the CEM weights range from 0.07 to 5.29, and in the high cSES population, the weights range from 0.33 to 4.00.

Similarly, there is no difference in prevalence of elevated depressive symptoms among wives of veterans compared to wives of non-veterans in the pooled sample, or either cSES subgroup, however, point estimates suggest husband’s GI Bill eligibility may be protective for wives’ odds of elevated depressive symptoms in a larger sample (OR = 0.55, 95%CI: 0.14, 2.26), p = 0.410) (Table 3). Interaction analyses show no difference in the relationship between husband’s GI Bill eligibility and odds of elevated depressive symptoms by cSES (OR = 0.81, 95%CI: (-0.85, 2.47), p = 0.338).

**Table 3:** Results for odds of elevated depressive symptoms among GI Bill eligible veteran wives compared to non-veteran wives.

	Model 1			Model 2		
	OR	95% CI	p	OR	95% CI	p
Whole Population	0.91	(0.44, 1.90)	0.807	1.24	(0.55, 2.79)	0.605
Low cSES	0.55	(0.14, 2.26)	0.410	0.93	(0.16, 5.37)	0.934
High cSES	1.10	(0.44, 2.73)	0.836	2.12	(0.73, 6.15)	0.167

Model 1: model includes birth year (continuous) and self-rated childhood health (indicator variables for very good, good, fair poor) because these variables were coarsened to facilitate matching

Model 2: Model 1 with additional adjustment for respondent’s education (linear spline)

Five observations were dropped in the low cSES model because both fair (2 observation) and poor (3 observations) childhood health perfectly predicted not having elevated depressive symptoms.

In sensitivity analyses for number of depressive symptoms using father’s education to create childhood SES subgroups, there was no association between husband’s GI Bill eligibility and wives’ depressive symptoms in the pooled population, or either cSES subgroup. Results using father’s education to operationalize cSES were similar to those using mother’s education in the pooled population, but the father’s education results were closer to the null for the low cSES subgroup and further from the null in the high cSES subgroup (Table 4). Results using father’s education to operationalize cSES for odds of elevated depressive symptoms were substantively similar to results using mother’s education (Table 5).

**Table 4:** Results for number of depressive symptoms among GI Bill eligible veteran wives compared to non-veteran wives when cSES is dichotomized by father’s education.

	Model 1			Model 2		
	Beta	95% CI	p	Beta	95% CI	p
Whole Population	-0.19	(-0.61, 0.22)	0.363	-0.06	(0.48, 0.36)	0.786
Low cSES	0.02	(-0.98, 1.02)	0.967	0.01	(-1.10, 1.10)	0.993
High cSES	-0.29	(-0.69, 0.12)	0.161	-0.14	(-0.55, 0.26)	0.490

Model 1: model includes birth year (continuous) and self-rated childhood health (indicator variables for very good, good, fair poor) because these variables were coarsened to facilitate matching

Model 2: Model 1 with additional adjustment for respondent’s education (linear spline)

There were 321 individuals included in the analytic sample when cSES was dichotomized by father’s education (<8 years = low cSES, N = 96; > = 8 years = high cSES, N = 225).

**Table 5:** Results for odds of elevated depressive among GI Bill eligible veteran wives compared to non-veteran wives when cSES is dichotomized by father’s education.

	Model 1			Model 2		
	Beta	95% CI	p	Beta	95% CI	p
Whole Population	0.86	(0.41, 1.82)	0.698	1.22	(0.54, 2.73)	0.636
Low cSES	1.07	(0.35, 3.25)	0.901	1.33	(0.36, 4.96)	0.668
High cSES	0.64	(0.20, 1.98)	0.433	1.02	(0.29, 3.59)	0.973

Model 1: model includes birth year (continuous) and self-rated childhood health (indicator variables for very good, good, fair poor) because these variables were coarsened to facilitate matching

Model 2: Model 1 with additional adjustment for respondent’s education (linear spline)

There were 321 individuals included in the analytic sample when cSES was dichotomized by father’s education (<8 years = low cSES, N = 96; > = 8 years = high cSES, N = 225).

In analyses examining markers of adult SES, veteran wives reported 0.6 more years of education than non-veteran wives in the pooled population (95%CI: (0.19, 1.01), p = 0.004), 0.95 more years of education in the low cSES subgroup (95%CI: (0.07, 1.83), p = 0.034), and 0.45 more years of education in the high cSES subgroup (95%CI: -0.01, 0.91), p = 0.054), though high cSES results were not statistically significant at the traditional type I error rate of 5%. Similarly, in the pooled population veteran wives reported $8,710 higher income per capita than non-veteran wives (95%CI: (390, 17,031), = 0.040), and in the low cSES group veteran wives reported $11,771 higher income per capita than non-veteran wives (95%CI: 1,558, 21,983), but there was no difference among high cSES veteran and non-veteran wives. There was no difference in wealth per capita between veteran and non-veteran wives in the pooled population or either cSES subgroup (Table 6).

**Table 6:** Difference in SES markers between veteran wives and non-veteran wives.

	Years of Education			Income per capita in 2010			Wealth per capita in 2010		
	Beta	95% CI	p	Beta	95% CI	p	Beta	95% CI	p
Whole Population	0.60	(0.19, 1.01)	0.004	8,710	(390, 17,031)	0.040	-104,231	(-304,071, 95,610)	0.306
Low cSES	0.95	(0.07, 1.83)	0.034	11,771	(1,558, 21,983)	0.024	13,583	(-266,867, 294,032)	0.924
High cSES	0.45	(-0.01, 0.91)	0.054	8,946	(-1,891, 19,783)	0.105	-123,878	(-379,392, 131,635)	0.341

The covariate adjustment (CA) low cSES analytic sample had 88 non-veteran wives and 34 veteran wives; veteran wives were slightly younger and more likely to be Non-Hispanic White than non-veteran wives. The high cSES CA sample included 170 non-veteran wives and 71 veteran wives; veteran wives were slightly younger and were more likely to know their father’s education than non-veteran wives. The propensity score matching (PSM) low cSES analytic sample contained 25 non-veteran wives and 34 veteran wives; veteran wives were less likely to report excellent child health and more likely to report childhood depression than non-veteran wives. The PSM high cSES analytic sample included 72 non-veteran wives and 71 veteran wives; veteran wives were less likely to have missing data on their father’s education than non-veteran wives (Table 7).

**Table 7:** Distribution of covariates in the CA and PSM analytic samples.

	Covariate Adjustment								Propensity Score Matching							
	Low cSES				High cSES				Low cSES				High cSES			
	Non-Veteran Spouses (N = 88)		Veteran Spouses (N = 34)		Non-Veteran Spouses (N = 170)		Veteran Spouses (N = 71)		Non-Veteran Spouses (N = 25)		Veteran Spouses (N = 34)		Non-Veteran Spouses (N = 72)		Veteran Spouses (N = 71)	
	N (mean)	% (sd)	N (mean)	% (sd)	N (mean)	% (sd)	N (mean)	% (sd)	N (mean)	% (sd)	N (mean)	% (sd)	N (mean)	% (sd)	N (mean)	% (sd)
Age in 2010	(80.1)	(4.2)	(76.6)	(3.3)	(80.2)	(4.1)	(76.8)	(2.1)	(77.8)	(3.9)	(76.6)	(3.3)	(77.9)	(2.8)	(76.8)	(2.1)
Non-Hispanic White	62	70.5	28	82.4	160	94.1	66	93.0	21	84.0	28	82.4	70	97.2	66	93.0
Non-Hispanic Black	13	14.8	4	11.8	8	4.7	4	5.6	3	12.0	4	11.8	2	2.8	4	5.6
Hispanic	12	13.6	2	5.9	2	1.2	1	1.4	1	4.0	2	5.9	0	0	1	1.4
Missing data on mother’s education	27	30.7	11	32.4	0	0	0	0	7	28.0	11	32.4	0	0	0	0
Missing data on father’s education	27	30.7	8	23.5	14	8.2	2	2.8	6	24.0	8	23.5	7	9.7	2	2.8
Childhood Health																
Excellent	47	53.4	14	41.2	76	44.7	29	40.8	17	68.0	14	41.2	34	47.2	29	40.8
Very good	20	22.7	13	38.2	57	33.5	26	36.6	4	16.0	13	38.2	26	36.1	26	36.6
Good	16	18.2	5	14.7	31	18.2	9	12.7	1	4.0	5	14.7	11	15.3	9	12.7
Fair	2	2.3	1	2.9	3	1.8	7	9.9	1	4.0	1	2.9	1	1.4	7	9.9
Poor	3	3.4	1	2.9	3	1.8	0	0	2	8.0	1	2.9	0	0	0	0
Depression	2	2.3	1	2.9	3	1.8	1	1.4	0	0	1	2.9	0	0	1	1.4

CA models indicating that veteran wives had fewer depressive symptoms than non-veteran wives were borderline significant (β = -0.41, 95%CI: (-0.87, 0.04), p = 0.075); there no difference in number of depressive symptoms between veteran and non-veteran wives in either subgroup, though all point estimates were in the same direction. In propensity score matching (PSM) models, there was no difference between veteran and non-veteran wives in the pooled population or among wives in the high cSES subgroup, however, in the low cSES subgroup veteran wives reported 1.1 fewer depressive symptoms than non-veteran wives, and the results were borderline significant (95%CI: (-2.37, 0.13), p = 0.080) (Table 8). CEM results were described above. Results for elevated depressive symptoms across all methodologies were null, however PSM results for the low cSES subgroup were trending towards significance (β = -0.16, 95%CI: (-0.38, 0.07), p = 0.171) (Table 9).

**Table 8:** Results from CA, PSM, and CEM methodologies for number of depressive symptoms.

	CA				PSM				CEM			
	N	Beta	95% CI	p	N	Beta	95% CI	p	N	Beta	95% CI	p
Whole Population	363	-0.41	(-0.87, 0.04)	0.075	210	-0.68	(-1.58, 0.21)	0.134	352	-0.23	(-0.62, 0.16)	0.253
Low cSES	122	-0.50	(-1.30, 0.29)	0.212	68	-1.12	(-2.37, 0.13)	0.080	95	-0.50	(-1.35, 0.35)	0.248
High cSES	241	-0.35	(-0.91, 0.21)	0.224	142	0.14	(-0.49, 0.77)	0.660	257	-0.10	(-0.55, 0.35)	0.667

In the PSM sample, the whole population included 193 weighted to 210 (weights range from 0.17–10), the low cSES sample included 59 weighted to 68 (weights range from 0.14–4), and the high cSES sample included 143 weighted to 142 (weights range from 0.07–9).

**Table 9:** Results from CA, PSM, and CEM methodologies for elevated depressive symptoms.

	CA				PSM				CEM			
	N	Beta	95% CI	p	N	Beta	95% CI	p	N	Beta	95% CI	p
Whole Population	363	-0.05	(-0.13, 0.04)	0.269	210	-0.09	(-0.26, 0.08)	0.286	352	-0.01	(-0.07, 0.06)	0.818
Low cSES	122	-0.07	(-0.22, 0.07)	0.336	68	-0.16	(-0.38, 0.07)	0.171	95	-0.06	(-0.21, 0.09)	0.430
High cSES	241	-0.05	(-0.15, 0.06)	0.375	142	0.04	(-0.07, 0.14)	0.497	257	0.01	(-0.06, 0.08)	0.823

Results from linear probability models.

In the PSM sample, the whole population included 193 weighted to 210 (weights range from 0.17–10), the low cSES sample included 59 weighted to 68 (weights range from 0.14–4), and the high cSES sample included 143 weighted to 142 (weights range from 0.07–9).

## Discussion

In this highly selected population of women married to the same man from 1955–2010, we found no evidence of a spillover of husband’s Korean War GI Bill eligibility for either number of depressive symptoms or odds of elevated depressive symptoms in pooled analysis, or in either cSES subgroup, however, results for the low cSES subgroup may have been statistically relevant in a larger sample. We found veteran wives had more education and income than non-veteran wives, and found results were not substantively different when father’s education was used to operationalize cSES rather than mother’s education.

While the HRS data are uniquely suited to answer lifecourse health questions, some important limitations must be acknowledged. First, we restricted these analyses to individuals who were alive in 2010, and were married to the same spouse from 1955–2010, meaning widows, women who died of any cause, and divorced women were all excluded from this sample. The bias induced by differential survival is difficult to gauge; if the GI Bill truly improved the recipients’ mental health, then the missing wives in the control group (e.g. due to suicide) would bias results toward the null. Second, due to differences in selection out of marriage and the analytic method used, these results have limited generalizability, however the CA and PSM results are applicable to a wider population (Tables 8 & 9). Third, similar to an intent-to-treat analysis, we studied Korean War GI Bill eligibility, not receipt of these benefits, potentially biasing our results towards the null. Fourth, residual confounding may be present in this observational study. Fifth, there was no clinical diagnosis of depression. Sixth, all data used in this study are self-reported. Finally, a large number of eligible women were missing data on the outcome and were therefore excluded from analysis (48%). Of the 411 women with missing outcome data, 380 (92.5%) were eligible for the low cSES control group; if women with more depressive symptoms were less likely to answer questions regarding depressive symptoms, non-depressed women would be over-representative in the low cSES control group, biasing these results towards the null. Despite these limitations, this is the first study, to our knowledge, examining the spillover effects of the Korean War GI Bill to wives of veterans, and represents an important contribution to the field.

There are at least two possible explanations for the null findings in this study. First, there may be no spillover effect of husband’s Korean War GI Bill eligibility to their wives’ mental health. Although GI Bill eligibility predicts fewer depressive symptoms among veterans than non-veterans [22], the mechanism underlying this relationship has not yet been elucidated. The mechanism may include factors that don’t directly spillover to wives, such as education or resilience to depression that was instilled during military training, which may explain the null finding for veteran’s wives. Second, this study is underpowered to detect an effect of the size observed in the low cSES subgroup; our matched sample size was sufficient to detect an effect size of 1.02 depressive symptoms at 80% power, or roughly double the effect size observed in this analysis. Additionally, we found low cSES veteran wives had more education and income than comparable non-veteran wives; SES is highly correlated with depression markers [3–6,8,38], further indicating that there may be an effect among low cSES veteran wives that this analysis was underpowered to detect.

In addition to our main analysis technique, CEM, we also present results for analyses using CA and PSM. CA is an approach commonly used in the public health literature where individuals with data on the exposure, outcome, and all of the matching covariates are included in the analytic sample, and little attention is paid to whether the exposed and unexposed groups are comparable; for example, the CA non-veteran wives in this analysis included more minorities, and more individuals with missing data on parent’s education than the veteran wives (Table 7), indicating that the unexposed group may be more socially disadvantaged than the exposed group.

CEM and PSM, on the other hand, are matching methods which attempt to reduce imbalance between the treatment and control groups across all the matching covariates by removing individuals for whom good matches do not exist in the available data. With PSM, the matching covariates are reduced to a single number, the probability of treatment, and individuals with similar probabilities for exposure are matched. With CEM, treatment and control units are matched directly on all the baseline covariates (or the coarsened distribution if the researcher chooses to coarsen). Non-matches are pruned from the data set (i.e. given a weight of zero), and the control units are weighted to create balance across the strata [37].

The contrast between the implementation of these two matching procedures results in a number of differences in the resultant analytic samples and the assumptions underlying the analytic samples. One important difference between these methods is their ability to approximate a randomized control trial by eliminating imbalance between the treatment and control groups on the observed covariates. With CEM, we know the matching variables are equally distributed between the treatment and control groups due to the matching mechanism (i.e. treatment and control groups are directly matched on all covariates); with PSM, the matching variables are assumed to be equally distributed based on the propensity score, but this may not always be the case. In fact, in head-to-head comparisons of the PSM and CEM, prior work has shown that CEM consistently outperforms PSM in terms of the covariate imbalance, particularly when a caliper is used for the propensity score matching [25,26]. Other strengths of CEM over PSM, include fewer assumptions [25], and more options for the treatment of missing data [39], however, the improved covariate balance, which results in better internal validity, is the main reason we have more faith in the CEM point estimates than the PSM results.

We found that husband’s eligibility for the Korean War GI Bill did not spillover to depression makers for veteran wives, even though veteran wives reported more education and income than non-veteran wives. Although these results were null, we suspect there is a true effect that our study was underpowered to detect. Future work should replicate these analyses in other data sets and continue to use rigorous methods to examine spillover effects of social policies, particularly when there are strong theoretical reasons to expect an association.
